# Clinical Features and 1-Year Prognosis of Patients with Suspected Sepsis and Positive Blood Cultures in the Emergency Department: A Single-Center Retrospective Study

**DOI:** 10.3390/medicina62071405

**Published:** 2026-07-20

**Authors:** Pietro Pozzessere, Maria Pascali, Vincenzo Brescia, Valeria Carrieri, Antonio Saracino, Giulia Vitariello, Roberto Lovero, Giuseppe Troiano, Marica Colella, Angela Pia Cazzolla

**Affiliations:** 1 Department of Emergency Medicine, University Hospital of Bari, 70124 Bari, Italy; pieropozzessere66@gmail.com (P.P.); valeria.carrieri@gmail.com (V.C.); toniosaracino@gmail.com (A.S.); giulia.vitariello@libero.it (G.V.); 2 Emergency Department, IRCCS Ospedale San Raffaele, Università Vita-Salute San Raffaele, 20132 Milano, Italy; mariapascali86@gmail.com; 3 Clinical Pathology Unit, AOU Policlinico Consorziale di Bari-Ospedale Giovanni XXIII, 70124 Bari, Italy; vincenzo.brescia@policlinico.ba.it; 4 Department of Medicine and Surgery, University LUM ‘Giuseppe Degennaro’, 70010 Casamassima, Italy; troiano@lum.it; 5 Department of Theoretical and Applied Sciences (DiSTA), eCampus University, 22060 Novedrate, Italy; marica.colella1@gmail.com; 6 Interdisciplinary Department of Medicine, Section of Microbiology and Virology, University of Bari, 70124 Bari, Italy

**Keywords:** blood culture, PCT, PICS, SOFA score, emergency department

## Abstract

*Background and Objectives*: Sepsis is a life-threatening condition characterized by organ dysfunction due to a dysregulated host response to infection. Blood cultures (BCs) remain the gold standard for diagnosing bloodstream infections, yet their positivity rate is limited and culture-negative sepsis remains poorly characterized. This study aimed to identify clinical and laboratory predictors of BC positivity in patients with suspected sepsis and to evaluate the prognostic implications of BC results on 28-, 90-, and 365-day survival. *Materials and Methods*: We retrospectively analyzed adult patients admitted to the Emergency Department of Policlinico di Bari (March 2023–December 2025) with sepsis or septic shock (Sepsis-3 criteria). After excluding contaminated cultures, 912 patients were included (602 culture-positive; 310 culture-negative) with a 1-year follow up. Predictors of BC positivity were assessed using logistic regression and ROC analysis; survival was evaluated with Kaplan–Meier curves and Cox models adjusted for age and Sequential Organ Failure Assessment (SOFA) score. We also evaluated time-to-positivity (TTP) for all blood culture isolates. *Results*: Culture-positive patients presented with greater initial severity, including higher rates of septic shock, elevated procalcitonin, CRP, lactate, and SOFA scores, and more frequent thrombocytopenia. Procalcitonin showed good discriminatory ability (AUC (area under the ROC curve) 0.785; cutoff 0.85 ng/mL; sensitivity 59.8%; specificity 83.8%). Key laboratory thresholds included a procalcitonin cutoff of 0.85 ng/mL, while CRP and platelet count did not show meaningful discriminatory cutoffs beyond their significant differences between culture-positive and culture-negative patients. Survival was consistently lower in culture-positive patients at 28 days (72.9% vs. 85.9%), 90 days (57.5% vs. 71.8%), and 365 days (44.3% vs. 61.2%). However, in adjusted Cox regression, BC positivity was not independently associated with 1-year mortality (HR 1.46; *p* = 0.092), whereas age and SOFA score remained significant predictors. *Conclusions*: Blood culture positivity identifies patients with greater initial severity, explaining their poorer unadjusted survival. However, culture positivity itself is not an independent predictor of long-term mortality, as age and organ dysfunction remain the main determinants of outcome. Procalcitonin showed solid discriminatory ability for true bacteremia.

## 1. Introduction

Sepsis is a life-threatening organ dysfunction caused by a dysregulated host response to infection [1]. Septic shock represents its most severe form and is associated with markedly increased mortality [2]. Globally, sepsis accounts for 48.9 million cases and 11 million deaths annually, representing 19.7% of all deaths [3,4,5]. Mortality remains high—20–40% in high-income countries and >50% in low-income settings—despite improvements in early recognition and standardized management [6,7].

Bacteria are the most frequently identified pathogens (63%), followed by fungi (19%) [8,9]. However, up to 42% of patients have no identifiable pathogen, likely due to viral or protozoan infections or suboptimal timing of blood culture collection [10,11,12,13,14,15]. Blood cultures remain the gold standard for diagnosing bloodstream infections and are recommended before antimicrobial therapy initiation [7,16,17,18]. Nevertheless, their positivity rate is limited, results require ≥48 h, and contamination is common [19,20,21,22,23].

Recent evidence shows that patients with culture-positive and culture-negative sepsis may have similar resource use and clinical outcomes [24,25], yet culture-negative sepsis remains poorly characterized [9,26]. Assessing illness severity is therefore essential for risk stratification, and the SOFA score is widely used for this purpose [27,28,29]. An acute increase of ≥2 points identifies sepsis and correlates with increased mortality [30,31].

Most studies have focused on severe sepsis or septic shock and short-term mortality, without evaluating the full spectrum of sepsis or long-term outcomes [32]. This gap underscores the need to better understand predictors of blood culture positivity and their prognostic implications across different time horizons.

The primary aim of this study was to identify clinical and laboratory predictors of blood culture positivity in patients with suspected sepsis at emergency department admission. Long-term survival analyses (28, 90, and 365 days) were performed as secondary exploratory outcomes, and their methodological limitations are explicitly acknowledged. Based on this primary objective, we specifically evaluated whether clinical or laboratory characteristics at emergency department admission could predict blood culture positivity.

## 2. Materials and Methods

### 2.1. Study Design

This is a retrospective case–control study conducted at a single center between March 2023 and December 2025 at the Emergency Department of the Policlinico di Bari University Hospital. The study received approval from the Ethics Committees of the Bari University Hospital (no. 5842: 12 February 2019) of the Policlinic of Bari (Italy), in compliance with the Helsinki Declaration. Each patient or their legal representative provided consent for the data to be processed.

Clinical data were collected from 674 adult patients (≥18 years) who accessed the emergency department during the study period and who received a diagnosis of sepsis/septic shock according to the Third International Consensus Conference (Sepsis-3). The data from these patients were compared with 310 patients with negative blood cultures who consecutively presented to the same emergency department during the same study period. Patients with viral or parasitic infections or tuberculosis were excluded.

### 2.2. Data Collection

The data examined included: age, sex, comorbidities, physiological parameters (blood pressure, heart rate, oxygen saturation, body temperature, respiratory rate, Glasgow Coma Scale), laboratory data collected upon admission to the emergency department (hemoglobin, white blood cells, platelets, creatinine, total bilirubin, INR, CRP, procalcitonin, serum lactate), need for noninvasive ventilation or vasoactive amines, microbiological culture results (causative organism, Gram stain, time to positivity), and any patient death with the corresponding survival time. Patients were classified according to the severity of the infection based on the Sepsis-3 criteria of the Third Consensus Conference, updated with the 2026 recommendations.

**Comorbidity**: Thyroid disorders included both hypo- and hyperthyroidism based on documented medical history or ongoing treatment; in our cohort, hypothyroidism was predominant. Arrhythmias included hyperkinetic rhythm disorders, mainly atrial fibrillation, atrial flutter, and supraventricular tachycardia. Diabetes mellitus was recorded as a documented medical condition in the patient’s history; information on glycemic control (controlled vs. uncontrolled) was not consistently available in this retrospective dataset. Chronic kidney disease (CKD) was defined as a pre-existing diagnosis of renal insufficiency. Baseline eGFR values were not uniformly available for all patients, preventing precise KDIGO staging.

**Severity Assessment (SOFA score)**: Patients were classified according to Sepsis-3 criteria, updated with the 2026 recommendations. Organ dysfunction was assessed using the Sequential Organ Failure Assessment (SOFA) score, calculated at ED admission based on six organ systems (respiratory, coagulation, liver, cardiovascular, central nervous system, renal). An acute increase of ≥2 points was used to identify sepsis.

All data collected refer to the time of admission to the emergency department.

**Blood Culture Collection and Microbiological Methods**: All consecutive patients for whom blood cultures were requested during the study period were considered for inclusion, provided that complete one-year follow-up data were available. Blood cultures were performed in the presence of clinical and/or laboratory suspicion of infection. In all patients, blood cultures were drawn before the administration of antimicrobial therapy, in accordance with Surviving Sepsis Campaign recommendations.

Two sets of blood cultures were collected from different venipuncture sites. Bottles were incubated in an automated continuous-monitoring system (BACT/ALERT VIRTUO Biomerieux Florence Italy). Organism identification was performed using MALDI-TOF mass spectrometry and standard biochemical methods. Time to positivity was recorded for all samples.

The initial diagnosis (infection, sepsis, septic shock) was established prospectively by ED physicians applying Sepsis-3 criteria. The “Absence of infection” and “Infection” categories refer to patients who underwent blood culture sampling for suspected sepsis but did not meet Sepsis-3 criteria upon retrospective review, reflecting the real clinical spectrum and avoiding spectrum bias.

Patients with a positive culture within five days were classified as culture-positive (CP). Those with no identifiable pathogen after five days were classified as culture-negative (CN). Bloodstream infection was not considered synonymous with sepsis, and blood culture positivity alone was not used as a surrogate for true bacteremia. Microbiological results were interpreted within the clinical context to distinguish true bloodstream infection from contamination.

**Blood culture contamination criteria**: Blood cultures were classified as contaminated when typical skin commensals (coagulase-negative staphylococci, *Corynebacterium* spp., *Cutibacterium* spp., *Micrococcus* spp.) grew in a single bottle or single set, without clinical evidence of systemic infection. Contaminated cultures were excluded when one or more of the following criteria were met:

➢ negative inflammatory markers (normal CRP and PCT ≤ 0.09 ng/mL);
➢ absence of clinical signs of sepsis;
➢ time to positivity > 48 h;
➢ discordant results across bottles/sets;
➢ presence of an alternative diagnosis explaining the initial presentation.

Conversely, coagulase-negative staphylococci included in the culture-positive (CP) group were classified as true bacteremia when they met at least one of the following criteria: **≥2 positive bottles/sets, time to positivity < 24 h**, or **clear clinical correlation** (sepsis or septic shock, elevated inflammatory markers, intravascular devices, or a plausible infectious focus).

### 2.3. Statistical Analysis

Continuous variables were expressed as median and interquartile range (IQR) or mean ± standard deviation (SD), as appropriate. Continuous variables of the culture-positive (CP) and culture-negative (CN) groups were compared using the Mann–Whitney test. Categorical variables were compared using Pearson’s χ^2^ test or Fisher’s exact test when appropriate. Because infection site comprised multiple categories, an overall χ^2^ test was first performed; after a significant global result, pairwise comparisons were carried out using Fisher’s exact test, with *p*-values adjusted using the Holm–Bonferroni procedure.

Comparison of time to positivity among different pathogens was performed using the Kruskal–Wallis test, followed by Dunn’s post hoc multiple comparison test with Holm correction. Pathogens represented by fewer than five isolates were excluded from inferential analyses because of insufficient sample size but were included in descriptive analyses with median and IQR reported.

To identify predictors of blood culture positivity, univariate logistic regression was performed, with results expressed as odds ratios (ORs) and 95% confidence intervals (CIs). Multivariate logistic regression models were then fitted to identify independent predictors. Continuous variables were retained on their original scale without categorization, and variable inclusion was based on clinical relevance rather than automated selection procedures. Model discrimination was assessed using receiver operating characteristic (ROC) analysis.

The initially reported AUC (area under the ROC curve) was an **apparent AUC**, derived from the full dataset without training/test split. Internal validation was performed using **bootstrap resampling** with **2000 replicates**, yielding a mean bootstrap AUC of 0.784 (95% CI 0.758–0.840) and negligible optimism (−0.0006). Harrell’s bootstrap optimism-correction approach (500 replicates) produced an optimism-corrected AUC of 0.786, confirming the internal stability of the model. Model calibration was evaluated using the Hosmer–Lemeshow test [33,34].

To avoid redundancy and multicollinearity, individual SOFA components were not included together with the composite SOFA score. A **sensitivity analysis** was performed replacing the composite SOFA score with its individual components to assess their contribution to culture positivity.

Survival analysis was conducted using the Kaplan–Meier method. Comparisons between survival curves were performed using Cox regression analysis, with results expressed as hazard ratios adjusted for relevant covariates. For the survival analysis, no patient was lost to follow-up. All individuals had complete outcome information at 28, 90, and 365 days. Censoring occurred exclusively at the administrative end of the observation period and was therefore considered non-informative, as it was unrelated to clinical status or disease severity. The median follow-up time corresponded to the full 365-day observation window in both groups. Statistical significance was set at *p* < 0.05. The MedCalc^®^ (11.6.1.0) program was used for statistical analysis.

## 3. Results

During the observation period, 984 consecutive patient cases with 1-year follow-up were analyzed, of whom 674 (68.49%) had positive blood cultures (PCs) and 310 (31.5%) had negative blood cultures (CN). Of the 674 patients, 72 had blood cultures suspected of contamination and were excluded from the analysis.

Blood cultures were considered contaminated if coagulase-negative staphylococci were detected in the presence of negative inflammatory markers (both CRP and PCT, or PCT ≤ 0.09 at the time of blood culture collection or during follow-up) and an alternative diagnosis to sepsis was identified to explain the presenting symptoms.

Therefore, 912 patients were included, of whom 602 (66%) had positive blood cultures and 310 (34%) had negative blood cultures. Continuous and categorical variables were compared between the two groups. Continuous variables were expressed as medians and interquartile ranges (IQR) and compared using the Mann–Whitney test. Categorical variables were expressed as percentages with 95% confidence intervals (95% CIs) and compared using the chi-square test or Fisher’s exact test when appropriate. A *p*-value < 0.05 was considered statistically significant (Table 1).

Patients with positive blood cultures (CP) showed higher illness severity, as reflected by higher SOFA scores, more frequent need for vasopressors, and elevated lactate levels, than patients with negative blood cultures or contaminants (CN + CC). The most statistically significant differences concern biomarkers of sepsis and inflammation, with significantly higher levels of procalcitonin and CRP in CP patients. Furthermore, the CP group shows a significant increase in SOFA score and lactate levels, suggesting greater organ involvement and more marked hemodynamic instability. From a laboratory standpoint, CP patients present lower hemoglobin and platelet values and higher creatinine, bilirubin, and INR values, indicative of a more severe systemic condition. From an anamnestic standpoint, the CP group also presents a higher prevalence of renal, arrhythmic, hepatic, and neoplastic disease. Finally, the greater use of vasoactive amines in the CP group confirms the presence of greater circulatory instability. Overall, positive blood cultures are associated with a higher infectious burden, increased clinical severity, and greater multiorgan dysfunction.

The CP and CN groups were generally comparable for most clinical variables considered. No statistically significant differences emerged for age or most comorbidities. Among comorbidities, the presence of renal disease appears to significantly influence positive blood cultures, highlighting how renal disease could lead to a decline in immune defenses and a higher circulating bacterial load.

Patients were classified according to the severity criteria defined by the latest definition of sepsis in patients without infection, with infection, sepsis, and septic shock.

The characteristics of the sample studied are shown in Table 2.

The CP group showed a significantly more severe distribution of clinical severity compared to the CN group. Specifically, septic shock was significantly more frequent in CP patients (15.1% vs. 3.5%), while sepsis was the predominant category in both groups, with a higher prevalence in CP patients (58.5% vs. 50.3%). Conversely, the “no infection” and “infection” categories were relatively more frequent in the CN group. Overall, these data suggest a strong association between positive blood cultures and greater clinical severity, with a significant increase in the most severe forms of sepsis, particularly septic shock.

The microbiological profile showed a predominance of Gram-negative bacteria, with *E. coli* and *K. pneumoniae* being the most frequently isolated species, accompanied by a high presence of coagulase-negative staphylococci. Pathogens with intermediate frequency included *Enterococcus* spp., *Pseudomonas aeruginosa*, and *Proteus mirabilis*, a pattern suggesting a significant contribution from healthcare-associated infections.

Fungal isolates, primarily *Candida albicans*, were less common but still clinically significant. The organisms were classified according to the characteristics of Gram-positive strains. The overall percentages exceeded 100% because polymicrobial infections were present in several cases.

Overall, Gram-positives constituted the largest group, while Gram-negatives, particularly Enterobacteriaceae, represented a substantial portion of the isolates. Fungal infections, however, were sporadic.

In the study, various pathogens were isolated from positive blood cultures. The most frequently detected microorganisms are listed in Table 3.

Table 3 reports the total number of isolates, including polymicrobial cultures; therefore, the sum of isolates exceeds the number of patients. In contrast, Table 4 reports species-level data based on the 602 patients with positive blood cultures.

In the analyzed sample, a clear prevalence of Gram-positive microorganisms was observed, representing approximately two-thirds of the total isolates. Gram-negative bacteria constituted just over a third of the isolates, while fungal infections were relatively rare (1.6%), mainly caused by opportunistic fungal species. This distribution suggests an epidemiological predominance of Gram-positive bacteria in the bacteremia analyzed. From a clinical perspective, these results may have important implications for the choice of initial empiric antibiotic therapy, supporting the need for adequate coverage against Gram-positive pathogens in patients with suspected bacteremia.

The three most common pathogens were: [Table 4] *Escherichia coli* (128 isolates, 21.3%), *Staphylococcus epidermidis* (80 isolates, 13.3%), and *Staphylococcus hominis* (81 isolates, 13.5%).

Overall, these three microorganisms accounted for almost half of all isolates. They were followed by *Staphylococcus aureus* (69; 11.5%) and *Klebsiella pneumoniae* complex (51; 8.5%). The remaining bacterial isolates were grouped in the table under the “Other” category, which includes less frequently identified microorganisms and which together accounted for 4.8% of the total.

These data confirm the high incidence of Gram-positive pathogens (370 isolates, 61.5%) versus Gram-negative ones (221 isolates, *36.7*%).

The microbiological distribution of pathogens isolated in patients with positive blood cultures (PCs) was analyzed. The percentages were calculated based on the total number of PC patients (*n* = 602).

In the sample of patients with positive blood cultures, a predominance of Gram-positive cocci, particularly coagulase-negative staphylococci, was observed, along with a significant presence of Gram-negative enterobacteria. The most frequently isolated pathogen was *Escherichia coli* (21.3%), followed by *Staphylococcus hominis*, *Staphylococcus epidermidis*, and *Staphylococcus aureus*. Among the most clinically relevant Gram-negative bacteria, *Klebsiella pneumoniae* complex, *Pseudomonas aeruginosa*, and *Proteus mirabilis* were also observed. Fungal infections were relatively rare, predominantly *Candida albicans*. Overall, the observed microbiological profile suggests a prevalence of bacteremia caused by staphylococci, enterobacteria, and nosocomial opportunistic pathogens, consistent with a population with high clinical and healthcare complexity.

The mean time for blood cultures to become positive was 19.6 h ± 11.2 h.

**Figure 1:**
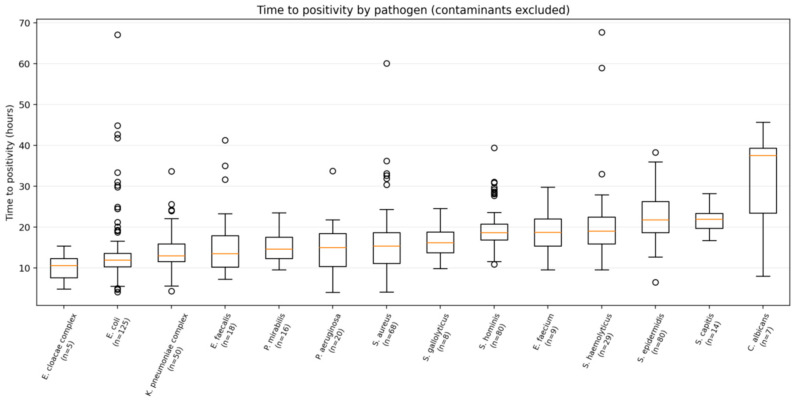
Boxplots show median, interquartile range, and outlying values. Enteric Gram-negative organisms showed shorter median TTP values, whereas coagulase-negative staphylococci and *Candida albicans* showed longer median TTP values.

Significant differences in time to positivity were observed across pathogens (Kruskal–Wallis H = 170.06, *p* < 0.001). Pathogen-level comparisons were restricted to microorganisms with at least 5 isolates and available time-to-positivity (TTP) data. *Enterobacter cloacae* complex, *Escherichia coli*, and *Klebsiella pneumoniae* complex showed the shortest median TTP values (10.6–12.9 h), whereas coagulase-negative staphylococci showed longer median values (18.7–21.9 h). Dunn post hoc testing with Holm correction confirmed significantly shorter TTP values for *Enterobacterales* and *Staphylococcus aureus* compared with several coagulase-negative staphylococci. This supports the clinical interpretation that TTP is strongly pathogen-dependent and that early blood culture positivity is more typical of rapidly growing bloodstream pathogens such as enteric Gram-negative bacteria. Pathogens with shorter TTP were more frequently isolated in patients with higher SOFA scores and greater hemodynamic instability, suggesting that rapid positivity reflects higher circulating bacterial burden. Shorter TTP was also associated with higher unadjusted mortality at 28 and 90 days, although this association was attenuated after adjustment for age and SOFA score (Figure 1).

Table 5 shows that the distribution of infection sites differed between culture-positive and culture-negative patients. Categorical variables were compared using Pearson’s χ^2^ test. Because infection site comprised multiple categories, an overall χ^2^ test was first performed. After a significant global result, pairwise comparisons were carried out using Fisher’s exact test. *p*-values were adjusted for multiple testing using the Holm–Bonferroni procedure. The ‘Blood’ category (primary bloodstream infection) was excluded from inferential testing because it is structurally absent among patients with negative blood cultures. In the revised classification, the ‘Blood’ category is now explicitly defined as primary bacteremia, i.e., bloodstream infection without an identifiable anatomical focus. Following re-evaluation of the dataset, 73 patients in whom alternative infectious sources could not be excluded were reassigned to the ‘Unknown origin’ category to improve classification accuracy.

The overall distribution of infection sites differed significantly between patients with positive and negative blood cultures (χ^2^ = 100.53, df = 11, *p* < 0.001). After Holm correction for multiple comparisons, lung infection, infective endocarditis (heart), and fever without an identified source remained significantly associated with blood culture positivity. Gallbladder infection lost statistical significance after adjustment, whereas intestinal infection showed borderline significance (adjusted *p* = 0.052). No statistically significant associations were observed for urinary tract, bone, skin, brain, unknown origin, or other infection sites.

This finding is consistent with current understanding of the pathophysiology of pulmonary infections. Pneumonia is confined to the lung parenchyma, and hematogenous dissemination occurs only in a minority of cases. Pneumonia is caused by pathogens that are infrequently recovered from blood cultures, including atypical bacteria and respiratory viruses. These mechanisms explain why lung infections show a significantly lower proportion of positive blood cultures. Pneumonia is typically confined to the lung parenchyma, hematogenous dissemination occurs only in a minority of cases, and many etiologic agents—such as atypical bacteria and respiratory viruses—are rarely recovered from blood cultures. Therefore, the markedly lower rate of blood culture positivity in pulmonary infections observed in our cohort is physiologically expected and consistent with current evidence.

From the significance of the tests, it is clear that the most evident result is that lung infections rarely present positive blood cultures and therefore the search for the pathogen should be carried out through targeted tests on respiratory samples, such as sputum or bronchoalveolar lavage cultures and, when indicated, urinary antigen tests or molecular panels on respiratory samples, as reported in the literature [35,36].

To evaluate the association between clinical and laboratory variables and the probability of positive cultures, a univariate analysis using logistic regression was performed. The results are expressed as Odds Ratios (OR) with 95% confidence intervals (95% CI). A *p*-value < 0.05 was considered statistically significant (Table 6).

The inclusion of age and sex in the univariate analysis did not reveal a statistically significant association with positive blood culture results. Specifically, age did not show any significant increase in the risk of a positive blood culture; male sex was not associated with a greater likelihood of bacteremia. Conversely, the following remained significantly associated with positive blood culture results: increased procalcitonin, CRP, lactate, and SOFA; presence of renal, hepatic, arrhythmic, and neoplastic disease; and need for vasoactive amines. Of all the variables analyzed, the use of vasoactive amines was the strongest association with positive blood culture results, suggesting a close correlation between bacteremia and hemodynamic instability.

The results of the univariate analysis were considered the variables to be included in the multivariate analysis, along with the most important clinical variables, to identify those that independently influence positive blood culture results. To avoid data redundancy, the indicators present in the SOFA score were excluded [Table 7].

In the multivariate model, a positive blood culture was independently associated with procalcitonin (OR 1.60, *p* = 0.006). The SOFA score was borderline associated (aOR 1.14, *p* = 0.051), and there was a trend toward significance. CRP, lactate, liver disease, malignancy, and arrhythmic and renal disease did not maintain independent statistical significance in the final model. The model showed good overall discriminatory ability (Likelihood Ratio Test *p* < 0.001, AUC 0.819) and suggests that increased clinical severity and infectious burden are the main independent determinants of positive blood culture results. Collinearity analysis showed no clinically relevant collinearity, with generally low VIF values. The model’s AIC was 233.11, suggesting a good balance between predictive accuracy and model complexity.

The initially reported AUC of the multivariate logistic regression model represented an **apparent AUC**, as it was calculated on the full dataset without training/test split. To assess the internal stability of the model, **bootstrap validation** was performed using **2000 resamples**, yielding a mean bootstrap AUC of **0.784** (95% CI 0.758–0.840). The estimated optimism was negligible (−0.0006), indicating absence of overfitting. In addition, **Harrell’s bootstrap optimism-correction approach** (500 replicates) produced an optimism-corrected AUC of **0.786**, confirming the robustness of the model’s discriminatory performance.

Model calibration was acceptable (Hosmer–Lemeshow *p* = 0.322), and collinearity analysis showed no clinically relevant multicollinearity (all VIF < 5). Continuous variables were retained on their original scale, and variable inclusion was based on clinical relevance rather than automated selection procedures.

To address potential redundancy between the composite SOFA score and its individual components, a **sensitivity analysis** was performed, replacing the composite SOFA score with its individual organ dysfunction variables. This analysis showed that **cardiovascular and respiratory dysfunction** were the main contributors to the association with blood culture positivity, without altering the overall conclusions of the model.

**Figure 2:**
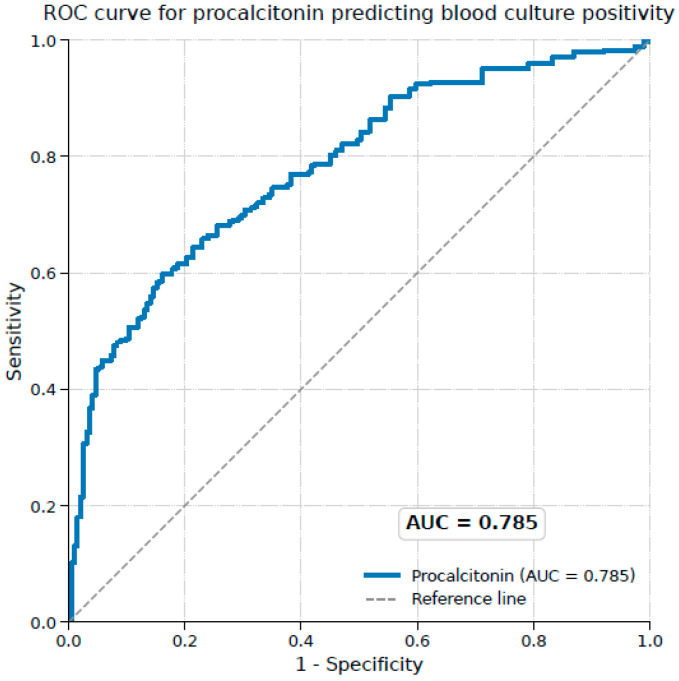
Receiving operating characteristic (ROC) curve of procalcitonin for predicting clinically significant blood culture positivity. The area under the ROC curve (AUC) is reported in the figure.

To evaluate the discriminatory ability of procalcitonin (PCT) in distinguishing patients with positive blood culture from the group of patients with negative blood culture, a ROC analysis was performed (Figure 2).

ROC analysis demonstrates that procalcitonin has good discriminatory ability in predicting blood culture positivity. An AUC of 0.785 (IC 95%, 0.748–0.826, according to the De Long method) suggests a moderate–high diagnostic performance, significantly superior to chance (AUC = 0.50). The optimal cutoff identified by the Youden index is 0.85 ng/mL (Youden’s index 0.375), with a sensitivity of 59.8% and specificity of 83.8% (Positive Predictive Value (PPV) 86.9%, Negative Predictive Value (NPV) 53.7%). From a clinical perspective, these results indicate that elevated procalcitonin levels significantly increase the likelihood of true bacteremia. The high specificity also suggests that values above the identified cutoff are particularly useful in supporting the diagnostic suspicion of systemic bacterial infection. However, the moderate sensitivity indicates that low procalcitonin values do not completely rule out the presence of bacteremia.

The model demonstrated fair to good discriminatory performance, with an AUC of 0.785 (95% CI: 0.7488–0.826), indicating stable and clinically relevant predictive performance.

At the optimal cutoff value of 0.85 ng/mL (n.v. < 0.05), corresponding to a Youden index of 0.375, the model achieved a sensitivity of 59.8% and a specificity of 83.8%. This profile suggests moderate discriminatory performance, characterized by greater accuracy in correctly excluding unaffected patients than in completely identifying positive cases.

**Figure 3:**
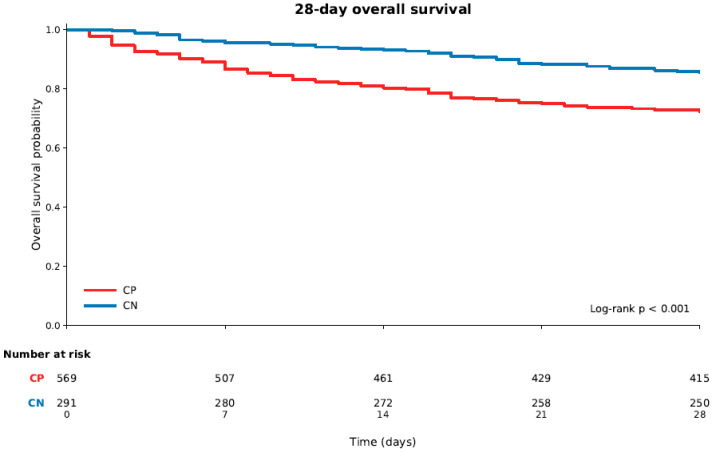

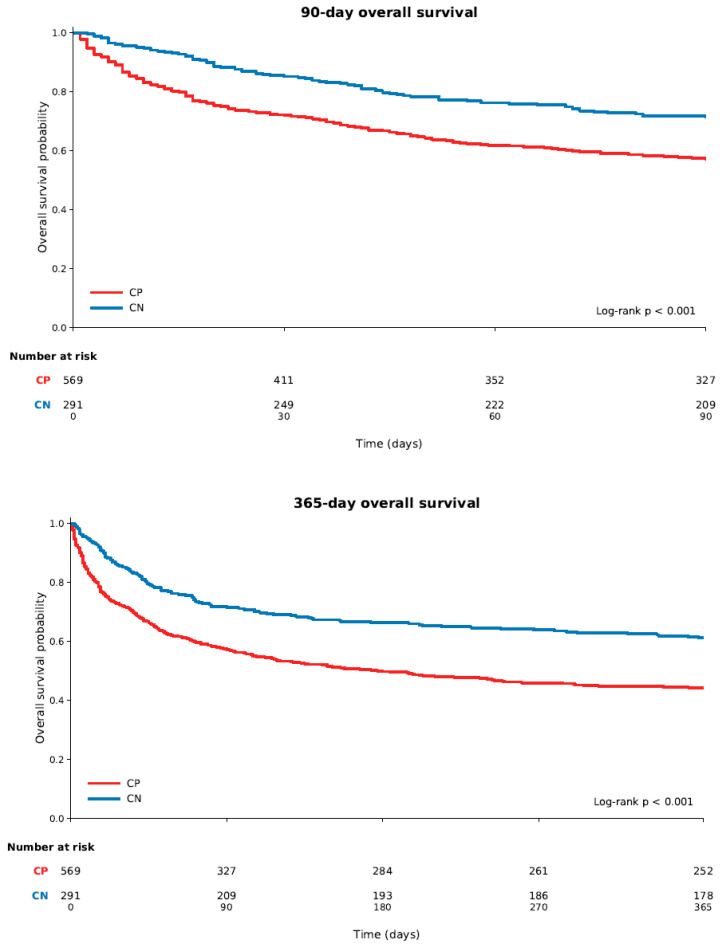
Kaplan–Meyer survival curves for 28-, 90-, and 365-day overall survival according to blood culture status (CP vs. CN). Survival distributions were compared using the log-rank test. Numbers at risk are shown below each plot.

A secondary objective of the analysis was to evaluate whether culture positivity affects survival in our population. Survival analysis was conducted using the Kaplan–Meier method (Figure 3).

Curves were compared using the log-rank test. A *p*-value < 0.05 was considered statistically significant. Only patients with available follow-up were included. No patient was lost to follow-up; therefore, all included subjects contributed complete survival data up to 1 year. Censoring was exclusively administrative and non-informative. The median follow-up time was 365 days in both groups. Accordingly, the survival analysis was performed on 569 patients.

Among patients with positive blood cultures, 415 of 569 (72.9%) survived to 28 days, 327 (57.5%) survived to 90 days, and 252 (44.3%) survived to 1 year. Among patients with negative blood cultures, 250 of 291 (85.9%) survived to 28 days, 209 (71.8%) survived to 90 days, and 178 (61.2%) survived to 1 year. Categorical variables were compared using Fisher’s exact test. (Table 8)

Clinically, these data suggest that a positive blood culture represents an important negative prognostic marker. It is associated with greater clinical severity, increased infectious burden, and worse short- and long-term outcomes.

Analysis of the survival curves confirmed a significant difference between patients with positive cultures (CP) and those with negative cultures (CN) at 28, 90, and 365 days (log-rank *p* ≤ 0.001).

Therefore, positive blood cultures identify patients with higher mortality from the outset, because the damage is early and non-progressive.

A multivariable Cox proportional hazards regression model was performed to evaluate independent predictors of mortality. The following covariates were included in the model: Positive blood culture status (CP), Age, SOFA score, log(Procalcitonin + 1), and Lactate. Outcome: overall survival.

Performing a Cox regression analysis to evaluate the association between positive blood cultures (CP) and 1-year mortality, the hazard ratio (HR) with 95% CI was 1.18 (0.92–1.52).

In a Cox regression analysis at 365 days, culture positivity was not independently associated with mortality after adjustment for age and SOFA score (HR 1.46, 95% CI 0.94–2.28, *p* = 0.092), while age and SOFA remained significant predictors (Table 9).

Therefore, we also assessed whether culture positivity was independently associated with mortality at 28 and 90 days.

Although Kaplan–Meier analysis showed a significant difference in survival between groups, this association was not confirmed after adjusting for age and SOFA score in the Cox regression model. The effect of culture positivity appears to be mediated by baseline severity rather than representing an independent prognostic factor. Log(PCT + 1) showed a statistically significant inverse association in the adjusted model for 28-day survival. The model demonstrated moderate discriminatory power, with a Harrell C index of 0.658.

Therefore, a positive blood culture identifies a group of septic patients with a worse prognosis but does not independently influence the mortality of these patients.

## 4. Discussion

Our retrospective study is the first to evaluate the influence of blood culture results, drawn within the first hour in an emergency department, on the prognosis of patients with positive or negative results at 1 year.

Our preliminary data show that patients with positive blood cultures present with greater initial clinical severity and, consequently, higher unadjusted mortality compared to patients with negative cultures.

After adjustment for age and SOFA score, blood culture positivity was not independently associated with mortality, indicating that the survival differences observed in the unadjusted analysis are fully explained by baseline disease severity rather than by bloodstream infection itself [37].

In our study, the only variable that could independently predict blood culture positivity was procalcitonin, although ROC analysis and the cutoff point indicated a greater ability to discriminate patients with negative blood cultures.

In our study, patients with positive blood cultures accounted for 66% of the overall population. This proportion, higher than that reported in previous studies on the same topic, reflects the fact that only patients with complete 1-year follow-up were included. Although not intentional, this resulted in a larger cohort of subjects with bacteremia and, indirectly, helped compensate for potential false positives due to contamination [38,39].

Some studies have evaluated the impact of positive blood cultures on the survival of patients with active infection. In a large retrospective study, Gupta et al. demonstrated that negative blood cultures represented an independent risk factor for short-term mortality in these patients. In fact, Gupta’s study was conducted using the National Patient Sample (NIS) and considered 6,843,279 cases of septic patients, of whom 47.1% had a negative blood culture, demonstrating that a negative blood culture was an independent prognostic factor for in-hospital mortality (OR, 1.75; 95% CI, 1.72–1.77) [38].

The results of this study confirmed what has been reported in the literature, as patients with positive blood cultures have a higher mortality rate than non-NC subjects (Table 8) [39,40,41,42,43,44].

Beyond these epidemiological observations, our cohort also suggests that specific comorbidities, particularly chronic kidney disease, may contribute to the biological vulnerability of culture-positive patients, offering a potential mechanistic explanation for the higher unadjusted mortality observed in this group. Renal disease emerged as a relevant comorbidity influencing blood culture positivity, suggesting that chronic nephropathy may contribute to impaired immune defenses and higher circulating bacterial burden [43]. A growing body of evidence indicates that patients with positive blood cultures have higher levels of endotoxinemia, and when this condition coexists with chronic kidney failure, the central role of the gut–kidney axis in amplifying systemic inflammation becomes increasingly evident. Gut microbiome dysbiosis, typical of chronic kidney disease, leads to increased intestinal permeability, bacterial translocation, and overgrowth of commensal microorganisms, with subsequent release of pro-inflammatory cytokines and uremic toxins such as p-cresol and indoxyl sulfate. These mediators sustain chronic low-grade inflammation, promote immunosenescence and sarcopenia, and ultimately aggravate the clinical vulnerability of septic patients [45,46,47,48,49,50,51].

Furthermore, time-to-positivity (TTP) provided additional insight into the biological behavior of bloodstream pathogens. Gram-negative *Enterobacterales* demonstrated markedly shorter TTP values, consistent with their rapid growth kinetics and higher circulating bacterial load. Conversely, coagulase-negative staphylococci showed longer TTP, reflecting lower bacteremia burden or borderline clinical significance. Shorter TTP was associated with greater organ dysfunction and hemodynamic instability, supporting its role as a marker of infection severity. Although shorter TTP correlated with higher unadjusted mortality, this association was largely mediated by baseline severity, as it weakened after adjustment for age and SOFA score. Despite the loss of statistical significance after adjustment, shorter TTP values may still hold clinical relevance. A rapidly positive blood culture can serve as an early warning sign of high bacterial burden and evolving severity, supporting closer monitoring or early ICU triage in selected patients. Conversely, longer TTP values—particularly in clinically improving patients—may assist clinicians in cautiously considering antibiotic de-escalation when aligned with microbiological and clinical data.

In line with previous observations, our study showed that Gram-negative pathogens reached blood culture positivity significantly earlier than Gram-positive organisms (12.6 h, IQR 10.7–15.5 vs. 18.7 h, IQR 14.4–22.3; Mann–Whitney U test, *p* < 0.001). This difference likely reflects the faster replication kinetics and higher circulating bacterial burden typically associated with Gram-negative infections [52,53].

Patients with positive blood cultures have significant differences in laboratory tests upon admission to the emergency department, especially thrombocytopenia, as well as sepsis markers (CRP and procalcitonin).

These data are consistent with the latest sepsis guidelines and several studies highlighting the correlation between reduced platelet counts and mortality in septic patients [54].

Platelet count trends in the early days of clinical development in septic patients represent an important prognostic indicator. Recent analyses have identified four distinct platelet response phenotypes, each associated with different outcomes.

Specifically, a failure to recover or a slow increase in platelet counts is associated with significantly higher mortality, reflecting persistent bone marrow dysfunction or platelet consumption driven by systemic inflammation and disseminated intravascular coagulation [55].

Conversely, a rapid normalization or increase in the platelet count is associated with a more favorable prognosis, suggesting a more effective recovery of hematological homeostasis and a more timely resolution of the inflammatory process [56,57].

Procalcitonin, a prohormone of calcitonin, encoded by the calc-1 gene on chromosome 11, is able to predict infections with reasonable accuracy and is recommended by sepsis guidelines for use as a biomarker of sepsis and, above all, as a parameter for assessing response to antibiotic therapy [58,59,60,61,62].

Finding a model that predicts bacteremia upon patient admission to the emergency department or a test that reduces the number of unnecessary blood cultures in the emergency room is the goal of numerous studies [62,63].

In this study, the population analysis was oriented towards identifying variables capable of accurately discriminating patients with bacteremia. To increase the model’s ability to identify predictors associated with positive blood cultures, a relatively higher proportion of patients with positive blood cultures compared to those with negative blood cultures was included. Among all clinical and laboratory variables analyzed, only procalcitonin showed a predictive role (*p* = 0.006) and an AUC of 0.785. The optimal cutoff of 0.85, with a sensitivity of 59.8% and a specificity of 83.8%, indicates that it is a more useful test for confirming a negative result than for predicting a positive result. Therefore, unlike what is reported in the literature, the data from this study indicate that procalcitonin cannot be used as a tool to avoid unnecessary blood cultures in the emergency department [64,65,66,67,68].

In interpreting the predictive model, several methodological aspects deserve consideration. The discriminatory performance originally reported represents an **apparent AUC**, as the model was fitted on the full dataset without a training/test split. To ensure transparency and reduce the risk of overfitting, we performed **bootstrap internal validation**, which confirmed the stability of the model (bootstrap AUC 0.784; 95% CI 0.758–0.840; negligible optimism). Harrell’s optimism-correction approach yielded similar results, supporting the robustness of the model’s discriminatory ability. These findings indicate that the predictive performance of procalcitonin is not inflated by model overfitting [33,34].

To avoid redundancy and multicollinearity, the composite SOFA score was used instead of its individual components. A sensitivity analysis replacing the composite score with individual organ dysfunction variables confirmed that cardiovascular and respiratory dysfunction were the main contributors to the association with blood culture positivity, without modifying the overall conclusions.

Finally, our modeling strategy followed a clinically driven approach, retaining continuous variables on their original scale and avoiding automated variable selection procedures. This aligns with recommended frameworks for transparent logistic regression model development and ensures methodological clarity. Nevertheless, external validation in independent cohorts remains necessary before the model can be applied in routine clinical practice [37,69,70,71,72,73,74].

In addition to these methodological considerations, it is important to acknowledge certain clinical variables that could not be incorporated into the predictive model. It is worth noting that rigors have been identified as a relevant clinical predictor of bacteremia in emergency department settings [75,76,77]. In our cohort, rigors were not systematically documented and could not be included among candidate predictors. This represents a limitation of the present model and highlights the need for prospective studies incorporating this variable.

While the primary aim of this study was to identify clinical and laboratory predictors of bacteremia at emergency department admission, short- and long-term mortality analyses were performed as a secondary exploratory objective. Given the retrospective nature of the study and the presence of uncontrolled confounders (e.g., antimicrobial timing, treatment heterogeneity, comorbidity burden), these prognostic findings should be interpreted cautiously and cannot be considered definitive prognostic evidence.

To contextualize these exploratory findings, we report the observed survival outcomes in culture-positive and culture-negative patients.

In our cohort, patients with positive blood cultures had higher unadjusted mortality; however, this association was not retained after adjustment for age and SOFA score [37,67,68,69,70,71,72,73,74]. Beyond the primary findings, an additional contribution of this study lies in the integration of short-term and long-term outcomes within a single real-world cohort of sepsis patients. While previous research has examined predictors of bacteremia and differences between culture-positive and culture-negative sepsis, few studies have extended follow-up to one year. In our analysis, the loss of statistical significance of blood culture positivity after adjustment for age and organ dysfunction provides clinically relevant insight: it suggests that the excess mortality observed in culture-positive patients is largely explained by baseline severity rather than by bacteremia itself. This interpretation reinforces the importance of early organ dysfunction assessment and offers a more nuanced understanding of the prognostic role of bloodstream infection in critical illness [43,71,72,73,74,75,76,77,78].

This finding is consistent with several studies that have documented that microbiologically confirmed bacteremia represents an unfavorable prognostic factor. In particular, Kang et al. demonstrated that blood culture positivity was an independent predictor of mortality in septic patients [69], while Leibovici et al. reported a higher mortality in patients with bacteremia compared to those with suspected infection but negative cultures [68]. Similar results were also observed in intensive care settings, where Rhee et al. highlighted a higher mortality in patients with sepsis and positive cultures compared to negative cultures [71,72,73], and Phua et al. confirmed a similar trend in a large multicenter Asian study [73].

However, it is important to note that not all studies are consistent. In some critically ill populations, as documented by the EPIC II study [70], mortality did not differ significantly between microbiologically documented infections and culture-negative clinical infections. Similarly, Bloos et al. observed that in patients with septic shock, mortality was more closely related to the severity of organ dysfunction than to culture positivity [74].

Overall, the results of this study rank among those highlighting a higher mortality associated with documented bacteremia, while acknowledging that the literature presents heterogeneous results depending on the clinical setting, the timing of antimicrobial therapy, and the characteristics of the population studied [75,76].

In this study, patients with positive blood cultures had a higher short-, medium-, and long-term mortality rate, greater multiorgan compromise, greater use of vasopressors and noninvasive ventilation, and a higher age.

In our unadjusted analysis, a positive blood culture was associated with increased mortality. However, this association lost significance after adjustment for age and SOFA score, indicating that the observed effect was likely mediated by the greater clinical severity of bacteremic patients rather than by culture positivity itself.

Our study also represents the first attempt to evaluate one-year mortality in patients with positive blood cultures. Although unadjusted analyses showed worse long-term outcomes, these differences were mediated by baseline organ dysfunction rather than by bloodstream infection itself. Long-term outcomes in survivors of critical illness have increasingly been framed within the concept of Post-Intensive Care Syndrome (PICS), a constellation of physical, cognitive, and psychological impairments that may persist after ICU discharge and represent a growing challenge for clinicians and healthcare systems [77,78,79]. Several predisposing factors described in the literature—including prolonged mechanical ventilation, deep sedation, delirium, ECMO support, and complications such as ARDS—have been associated with an increased risk of long-term deficits in ICU survivors [80,81,82,83]. The experience gained during and after the COVID-19 pandemic has further highlighted the relevance of these sequelae, with delirium emerging as one of the strongest predictors of cognitive decline and late mortality [84]. Overall, one-year mortality after severe critical illness commonly ranges between 20% and 40%, particularly in patients with advanced age, frailty, or multiorgan dysfunction, supporting the shift from the traditional concept of “ICU survival” to a broader focus on long-term outcomes [85]. In this context, the literature has proposed the notion of a “PICS biologic syndrome,” characterized by chronic inflammation, immunosuppression, and muscle catabolism, which may contribute to persistent vulnerability in selected patient populations [86,87,88,89]. In our study, although we did not collect functional, cognitive, or psychological outcomes, the observed late mortality among culture-positive patients is consistent with the known long-term fragility described in survivors of critical illness, without allowing any direct inference regarding PICS [90].

In our cohort, among patients with positive blood cultures, mortality between 90 days (the standard interval for defining PICS) and 1 year was observed in 98 subjects, including 10 (10.2%) with septic shock, 71 (72.4%) with sepsis, and 17 (17.3%) with documented infection. This finding suggests that bacteremia may represent a significant contributor to the biological and clinical vulnerability of survivors of critical illness [91].

This study has several limitations. No a priori sample size or power calculation was performed, as the study was based on a consecutive retrospective cohort. Furthermore, it is a retrospective, single-center study and, as such, has limited generalizability due to its dependence on the clinical setting, the population studied, and local practices. Antibiotic therapy and treatment timing were not considered in the assessment of variables that could influence mortality. Furthermore, potential contaminants were not considered among the pathogens, although the choice to consider a higher percentage of patients with positive blood cultures compared to negative ones was dictated by the possibility of filling this gap. In this regard, a greater bias toward patients with positive blood cultures may less accurately estimate the CN group with less stable ORs, but it has little impact on both ROC comparisons and, above all, Kaplan–Meyer and Cox regression analyses, due to the high number of events in both groups. Finally, rigors—an established clinical predictor of bacteremia—were not available in our dataset because they were not routinely documented in the electronic medical records. This omission may have limited the completeness of the predictive model and should be addressed in future prospective studies.

## 5. Conclusions

According to the results of our study, positive blood cultures collected in the emergency department identify a subgroup of septic patients with greater initial clinical severity, which accounts for their worse unadjusted short-, medium-, and long-term outcomes. The ability to predict positive culture results remains limited: among the available biomarkers, procalcitonin remains more useful as a confirmatory indicator of bacteremia than as a predictive tool.

The detection of bacteremia also significantly impacts short-term (28 days), intermediate (90 days), and long-term (1 year) survival. This trend reflects the greater initial severity of illness in culture-positive patients, which is known to influence long-term outcomes in survivors of critical illness.

## Figures and Tables

**Table 1:** Comparison of variables between patients with positive blood cultures (CP) and patients with negative blood cultures (CN). In particular, there is a statistically significant difference regarding arrhythmic pathology, renal pathology, liver pathology, presence of neoplasia, use of vasoactive amines, hemoglobin concentration, platelet count, bilirubin levels, PT INR, procalcitonin, CRP, SOFA SCORE, and lactate. These indices are increased more in the CP group than in the CN group.

Variable	CP	CN	*p*-Value
Age (median [IQR])	75 (64.0–84.0)	75 (59.0–84.0)	0.688 *
Male sex (%)	55.8% (52.2–59.6)	58.3% (53.7–66.7)	0.524 ***
Hypertension (%)	56.9% (52.2–59.6)	54.5% (49.9–63.1)	0.526 ***
Cardiac disease (%)	28.8% (24.6–31.3)	24.8% (19.2–30.8)	0.211 ***
Arrhythmic disease (%)	20.0% (16.7–22.7)	13.9% (6.9–15.3)	0.023 ***
Diabetes (%)	25.3% (20.6–27)	21.9% (19.7–31.3)	0.288 ***
Dyslipidemia (%)	20.0% (16.7–22.7)	20.6% (15.4–26.2)	0.862 ***
Dysthyroidism (%)	13.1% (10.1–15.1)	11.0% (6.8–13.7)	0.396 ***
Renal disease (%)	30.8% (25.1–31.9)	16.5% (12.1–22.2)	<0.001 **
Malignant neoplasm (%)	23.1% (18.7–24.9)	17.1% (13.3–23.7)	0.039 **
Hematological disease (%)	14.6% (10.4–15.5)	12.3% (10.1–17.6)	0.332 ***
Vascular disease (%)	15.5% (12.6–18)	11.6% (6.5–14.8)	0.132 ***
Liver disease (%)	9.5% (6.8–11.1)	5.2% (1.8–7.4)	0.028 ***
Neurological disease (%)	20.1% (17.0–23.0)	16.1% (12.3–18.5)	0.153 ***
Pulmonary disease (%)	21.3% (18.5–24.7)	21.6% (15.4–26.2)	0.932 ***
Noninvasive ventilation	16.6% (11.9–22.2)	15.6% (11.4–16.5)	0.705 ***
Vasoactive amines	23.5% (18.2–29.8)	8.1% (5.8–10.1)	<0.001 ***
Hemoglobin (g/dL)	11.1 (9.3–12.6)	11.9 (10.1–13.4)	<0.001 *
White blood cells (×10^3^/μL)	11.34 (7.49–16.00)	10.52 (8.03–15.68)	0.305 *
Platelets (×10^3^/μL)	203 (141.75–306.25)	259.50 (189.00–341.25	<0.001 *
Creatinine (mg/dL)	1.17 (0.77–1.93)	0.91 (0.67–1.42)	<0.001 *
Bilirubin (mg/dL)	0.79 (0.48–1.26)	0.62 (0.40–1.04)	<0.001 *
INR	1.07 (1.01–1.22)	1.04 (0.98–1.11)	<0.001 *
CRP (mg/L)	129.70 (51.85–228.60)	86.20 (36.15–154.70)	<0.001 *
Procalcitonin (ng/mL)	1.60 (0.29–12.62)	0.18 (0.08–0.53)	<0.001 *
SOFA SCORE	3.00 (1.00–5.00)	2.00 (1.00–4.00)	<0.001 *
Lactate (Time 0)	1.7 (1.10–2.90)	1.1 (0.8–1.5)	<0.001 *

* Mann—Whitney test; ** Fisher’s Exact Test; *** Chi-square.

**Table 2:** Characteristics of the sample studied. Percentage evaluation of the incidence of the described characteristics.

Classification	CN *n* (%)	CP *n*(%)	Total
No infection	47 (15.2%)	0	47
Infection	93 (30.0%)	149 (24.8%)	242
Sepsis	156 (50.3%)	352 (58.5%)	508
Septic shock	11 (3.5%)	91 (15.1%)	102
Missing	3 (1%)	10 (1.7%)	13
Total	310 (34%)	602 (66%)	912

**Table 3:** Prevalence of Pathogens in Patients with Positive Blood Cultures.

Category	N	%
Gram-pos bacteria	370	61.5
Gram-neg bacteria	221	36.7
Fungi	11	1.8
Total	602	100%

**Table 4:** Prevalence (%) of microorganisms identified in samples with positive blood cultures. Microorganisms were reported as absolute numbers (n) and percentages (%). Low-frequency isolates (<0.5%) were grouped under “Other microorganism” for clarity. Time-to-positivity (TTP) data were expressed in hours. IQR = interquartile range.

Microorganisms	Nr	%	Median TTP	IQR (h)
*Escherichia coli*	128	21.3%	11.95	(10.28–13.55)
*Staphylococcus* *hominis*	81	13.5%	18.66	(16.87–20.73)
*Staphylococcus* *epidermidis*	80	13.3%	21.75	(18.67–26.29)
*Staphylococcus* *aureus*	69	11.5	15.38	(11.08–18.64)
*Kl. pneumoniae complex*	51	8.5	12.95	(11.56–15.92)
*St. haemoliticus*	30	5.0	18.98	(15.90–22.48)
*Ps. Aeruginosa*	20	3.3	15.01	(10.32–18.39)
*Enterococcus faecalis*	18	3.0	13.52	(10.25–17.93)
*Proteus mirabilis*	17	2.8	14.62	(12.28–17.52)
*Staphylococcus capitis*	14	2.3	21.93	(19.70–23.32)
*Enterococcus faecium*	9	1.5	18.75	(15.35–22.03)
*Streptococcus gallolyticus*	8	1.3%	16.16	(13.75–18.83)
*Candida albicans*	7	1.2	37.50	(23.43–39.32)
*Enterobacter cloacae*	5	0.8	10.58	(7.60–12.32)
*Streptococcus agalactiae*	4	0.7	11.90	(9.24–12.98)
*Streptococcus dysgalactiae*	4	0.7%	10.85	(9.56–12.47)
*Streptococcus pneumoniae*	4	0.7%	12.07	(8.67–13.37)
*Streptococcus mitis/oralis*	4	0.7%	18.57	(16.65–22.15)
*Acinetobacter baumannii*	4	0.7%	11.11	(9.82–12.24)
*Streptococcus infantarius*	3	0.5%	12.28	(12.22–12.31)
*Streptococcus pyogenes*	3	0.5%	11.28	(10.28–19.25)
*Staphylococcus lugdunensis*	2	0.3%	22.54	(20.55–24.54)
*Streptococcus anginosus*	2	0.3%	26.53	(26.46–26.61)
*Stenotrophomonas maltophilia*	2	0.3	13.95	(12.98–14.92)
*Streptococcus salivarius*	2	0.3	12.58	(12.22–12.31)
*Staphylococcus cohnii* ssp. *cohnii*	2	0.3	19.80	(18.89–20.71)
Other single organisms	29	4.8		

Note: Species distribution is reported at the patient level (*n* = 602).

**Table 5:** Infection sites identified in the sample taken into consideration. Categorical variables were reported as absolute numbers and percentages.

Site	Positive Blood Cultures (CP)		Negative Blood Cultures (CN)		*p*-Value	Holm-Adjusted *p*
Lung	109	16.2%	79	48.5%	<0.001	<0.001
Urinary tract	74	11.0%	17	10.4%	0.85	1.00
Gallbladder	33	4.9%	2	1.2%	0.08	0.132
Heart	27	4.0%	0	0	<0.01	0.025
Bone	18	2.7%	3	1.8%	0.50	1.00
Skin	17	2.5%	6	3.7%	0.40	1.00
Intestine	15	2.2%	12	7.4%	0.01	0.052
Brain	14	2.1%	7	4.3%	0.20	0.895
Unknown origin	78	12.9%	30	18.4%	0.077	0.463
Other	4	0.6%	1	0.6%	1.00	1.00
Fever	0	0	6	3.7%	<0.01	<0.001
No information	87	12.9%	0	0	<0.001	

Overall χ^2^ = 100.53; degrees of freedom = 11; *p* = 1.40 × 10^−16^.

**Table 6:** Univariate analysis by means of linear regression of the clinical variables taken into consideration.

Variable	OR	95% CI	*p*-Value
Age	1.00	(0.99–1.01)	0.694
Male sex	0.91	(0.69–1.21)	0.525
Renal disease	2.26	(1.60–3.19)	<0.001
Arrhythmic disease	1.55	(1.06–2.27)	0.023
Liver disease	1.93	(1.09–3.41)	0.025
Malignant neoplasm	1.46	(1.03–2.07)	0.035
Vasoactive amines	3.48	2.21–5.45	<0.001
SOFA	1.13	1.07–1.20	<0.001
Lactate (time 0)	1.94	(1.49–2.54)	<0.001
Hemoglobin (g/dL)	0.88	0.83–0.94	<0.001
Platelets (×10^3^/μL)	0.998	0.996–0.999	<0.0001
Creatinine (mg/dL)	1.16	1.04–1.29	0.009
Bilirubin (mg/dL)	1.10	0.99–1.22	0.069
INR	1.23	0.97–1.57	0.092
CRP (mg/L)	1.004	(1.002–1.005)	<0.001
Procalcitonin (ng/mL)	1.02	1.01–1.04	<0.001

**Table 7:** Results of the multivariate logistic regression analysis to identify independent predictors of blood culture positivity. The aOR column reports the adjusted hazard ratios with the corresponding confidence intervals, while the VIF (Variance Inflation Factor) column describes the analysis of collinearity between the variables included in the model. VIF values < 5 indicate the absence of clinically relevant collinearity. The model was built by including the variables found to be significant in the univariate analysis and those that were clinically relevant, excluding the indicators already included in the SOFA score to avoid redundancy.

Variable	aOR	95% CI	VIF	*p*-Value
CPR (mg/mL)	1.00	(1.00–1.01)	2.5	0.498
Procalcitonin (ng/mL)	1.60	(1.14–2.24)	1.21	0.006
Renal disease	2.33	(0.97–5.60)	1.41	0.058
Liver disease	0.67	(0.15–2.96)	1.13	0.597
Arrhythmic disease	1.54	(0.68–3.47)	1.29	0.301
Malignant neoplasm	1.39	0.62–3.11	1.35	0.422
Lactate (T0)	1.37	(0.93–2.02)	2.41	0.109
SOFA	1.14	(1.01–1.31)	2.65	0.051

**Table 8:** Comparison of CP vs. CN Survival Days.

Days	CP nr (%)	CN nr (%)	CP Survival %	CN Survival %	Chi-Square	*p*-Value
28	569	291	72.9	85.9	9.238	<0.001
90	569	291	57.5	71.8	6.118	<0.001
365	569	291	44.3	61.2	9.254	<0.001

**Table 9:** Cox regression (HR adjusted for patient’s age and high-risk conditions) *.

Variable	Survival	HR (Adjusted)	95% CI	*p*-Value
CP	365 gg	1.46	0.94–2.28	0.092
	90 gg	1.49	0.89–2.49	0.133
	28 gg	1.84	0.90–3.76	0.092
Age	365 gg	1.03	1.01–1.04	<0.001
	90 gg	1.04	1.02–1.05	<0.001
	28 gg	1.02	1.00–1.05	0.023
SOFA	365 gg	1.15	1.08–1.23	<0.001
	90 gg	1.13	1.04–1.22	0.003
	28 gg	1.25	1.13–1.37	<0.001
Log(PCT + 1)	365 gg	0.94	0.83–1.07	0.367
	90 gg	0.95	0.82–1.10	0.481
	28 gg	0.78	0.63–0.97	0.024
Lactate (0)	365 gg	1.06	0.93–1.20	0.367
	90 gg	1.05	0.90–1.23	0.501
	28 gg	1.03	0.83–1.29	0.763

* In the Cox proportional hazards models, blood culture status was entered as a binary variable, with the CN group used as the reference category. Age, SOFA score, log-transformed procalcitonin, and lactate were included as continuous variables.

## Data Availability

The data presented in this study are available upon request from the corresponding authors.
